# Recent advances in prostatectomy for benign prostatic hyperplasia

**DOI:** 10.12688/f1000research.18179.1

**Published:** 2019-08-29

**Authors:** Alexis E. Te

**Affiliations:** 1 Weill Medical College of Cornell University, Ithaca, NY, USA; 2 Urology Program, Iris Cantor Men’s Health Center, 425 East 61st Street, 12th Floor, New York, NY, 10065, USA; 3 Brady Prostate Center and Urodynamics Laboratory, 525 East 68th Street, 9th Floor, New York, NY, 10065, USA

**Keywords:** BPH, LUTS, Transurethral Prostatectomy, Laser, Aquablation, Retrograde Ejaculation, Vapor Ablation, Thermotherapy, Electrovaporization, TURP, Open prostatectomy, Prostatic Stents, Stents, Benign Prostatic Hyperplasia, Lower Urinary Tract Symptoms, Robotic Prostatectomy

## Abstract

This review provides a brief overview of and commentary on currently available technology for the surgical treatment of obstructive benign prostatic hyperplasia causing lower urinary tract symptoms. This review provides references relevant to review and understand current technology that is clinically available.

In the last five years, advances in surgical options for obstructive benign prostatic hyperplasia (BPH) have progressed with new technologies and refinement of current options. These developments reflect the ongoing needs to still perfect this surgical approach. The most recent advancement is aquablation, the first non-thermal technology to resect prostate tissue; studies demonstrate efficacy and safety at least equivalent to those of transurethral resection of the prostate (TURP) in glands up to 150 mL and improved outcomes in retrograde ejaculation ^1– 4^.

As intermediate options to medical therapy and TURP, both prostatic urethral lift and vapor ablation are generally office procedures that demonstrate superior safety profiles, especially in regard to sexual function, and that have acceptable efficacy compared with TURP ^5– 9^. In the past, most studies-based superiority of procedures on efficacy outcome parameters such as International Prostate Symptom Score and urodynamic parameters assessing obstruction relief as well as intraoperative and perioperative safety parameters such as bleeding, lengths of hospital stay, and infections. However, with the increased availability of novel and minimally invasive procedures, there is an increasing perception/awareness of these therapies as viable alternatives to medical therapy. Studies such as the BPH-6 studies in which a combination of quality-of-life parameters have increased the importance in outcome parameters that affect procedure choice such as retrograde ejaculation, need for catheterization, and recovery time have made what were technically less efficacious procedures more “superior” in these studies ^10^. As such, many novel and minimally invasive procedures, including various prostatic stents and new technologies to resect prostate tissue or relieve prostatic outlet resistance, are still being aggressively developed. Many of these novel techniques, such as prostatic urethral lift and vapor ablation, are also designed to be used in an outpatient or office setting with minimal or local anesthesia.

Not surprisingly, many currently used technologies, including many traditional therapies, have evolved. Because it removes the risk of dilutional hyponatremia by using normal saline as an irrigant, the standard TURP is now performed with bipolar technologies with equal efficacy and improved safety parameters ^11– 14^. The open prostatectomy has also evolved to be incorporated in many robotic prostatectomy procedures, although its overall complication rate and hospital stay are still much higher than those of many transurethral procedures ^15– 17^. The well-known laser-assisted enucleation of the prostate, holmium laser enucleation of the prostate (HoLEP), is one of the most well-studied procedures, demonstrating efficacy and safety superior to those of traditional open prostatectomy and TURP. However, its high learning curve has limited its widespread acceptance and utility ^18– 20^. Despite being initially limited to centers of excellence, it has undergone growing popularity due to the increasing number of trainees who have come from these centers of excellence as well as the improved technology of morcellation and instrumentation. The procedure has been in use since its initial introduction 20 years ago. However, laser procedures did not undergo durable popularity and widespread clinical utility until the introduction of high-power 532-nm laser technology, or “GreenLight” laser. Initially known as photoselective vaporization (PVP) and introduced as a viable technology with the first multicenter article in 2004 ^21, 22^, it is now the most common laser procedure in the world as a pure laser vaporization procedure. This technology has also evolved from an 80-Watt technology to a 180-Watt technology capable of vaporizing prostate tissue more efficiently and faster ^23^. Many studies have demonstrated the laser’s clear superior safety profile in anticoagulated patients, high risk patients (high American Society of Anaesthesiologists score or Charlson index) and in large prostates ^24– 28^. Whereas most “GreenLight” procedures use pure vaporization techniques for glands up at about 80 g, vapoenucleations techniques have been applied to glands up to 376 mL ^29^. Although there are many competing laser technologies, none has yet to compare in terms of ease of use, widespread utility, and short learning curve with a high safety profile.

Over the last decade, the superior outcome efficacy and durability of HoLEP in comparison with open prostatectomy have suggested a modification in technique to incorporate enucleation with various technologies. Not surprisingly, laser enucleation of the prostate with GreenLight has evolved to demonstrate better efficacy with a maintained safety profile, especially in very large glands. The enucleation technique has expanded to include thulium laser technology as well as bipolar electrovaporization technology ^30– 33^. The vaporization enucleation technique with GreenLight has allowed enucleation and completion of prostatectomy without a mechanical morcellator with outcomes superior to those of standard PVP techniques, especially in larger glands ^29^.

With the goal of preserving sexual function, the preservation of antegrade ejaculation has become an area of focus and interest with current evolving technologies. Studies with prostatic urethral lift and vapor ablation and aquablation incorporate ejaculation outcomes and demonstrate increased preservation of antegrade ejaculation compared with traditional techniques. Although preservation of bladder neck structures is often associated with preservation of antegrade ejaculation, especially in those with large intravesical middle lobes, the current modern approach is the preservation of paracollicular structures in laser, aquablation, and bipolar electrosurgical prostatectomy techniques ^34^.
